# Simultaneous Suppression of the Dendrite Formation and Shuttle Effect in a Lithium–Sulfur Battery by Bilateral Solid Electrolyte Interface

**DOI:** 10.1002/advs.201700934

**Published:** 2018-07-23

**Authors:** Ling Fan, Suhua Chen, Jingyi Zhu, Ruifang Ma, Shuping Li, Ramakrishna Podila, Apparao M. Rao, Gongzheng Yang, Chengxin Wang, Qian Liu, Zhi Xu, Lixia Yuan, Yunhui Huang, Bingan Lu

**Affiliations:** ^1^ School of Physics and Electronics Hunan University Changsha 410082 China; ^2^ Department of Physics and Astronomy Clemson Nanomaterials Institute Clemson University Clemson SC 29634 USA; ^3^ Department of Mechanical and Aerospace Engineering New York University Brooklyn NY 11201 USA; ^4^ Key Laboratory for Advanced Battery Materials and System (MOE) School of Materials Science and Engineering Huazhong University of Science and Technology Wuhan Hubei 43004 China; ^5^ School of Materials Science and Engineering Sun Yat‐sen University Guangzhou 510275 China; ^6^ 2D Material Technology Company Limited Wing Lok Street, Sheung Wan Hong Kong 999077 China; ^7^ Fujian Strait Research Institute of Industrial Graphene Technologies Jinjiang Fujian 362200 China

**Keywords:** bilateral solid electrolyte interfaces, dendrite growth, lithium sulfur batteries, lithium sulfur pouch cells, shuttle effect, sulfur graphite full cells

## Abstract

Although the reversible and inexpensive energy storage characteristics of the lithium–sulfur (Li‐S) battery have made it a promising candidate for electrical energy storage, the dendrite growth (anode) and shuttle effect (cathode) hinder its practical application. Here, it is shown that new electrolytes for Li‐S batteries promote the simultaneous formation of bilateral solid electrolyte interfaces on the sulfur‐host cathode and lithium anode, thus effectively suppressing the shuttle effect and dendrite growth. These high‐capacity Li‐S batteries with new electrolytes exhibit a long‐term cycling stability, ultrafast‐charge/slow‐discharge rates, super‐low self‐discharge performance, and a capacity retention of 94.9% even after a 130 d long storage. Importantly, the long cycle stability of these industrial grade high‐capacity Li‐S pouch cells with new electrolytes will provide the basis for creating robust energy dense Li‐S batteries with an extensive life cycle.

## 1 Introduction

The high energy density, low cost, and the environmentally friendly nature of Li‐S batteries make them attractive for use in automotive or stationary electrical energy storage applications.^1^, ^2^, ^3^, ^4^, ^5^, ^6^, ^7^ However, the severe shuttle effect and dendrite growth, and the inferior Coulombic efficiency that characterize these Li‐S batteries have greatly limited their lifespan.^4^, ^8^, ^9^, ^10^ Although much effort has been expended in the development of novel sulfur‐host electrodes,^11^, ^12^, ^13^, ^14^, ^15^, ^16^, ^17^, ^18^, ^19^, ^20^, ^21^, ^22^, ^23^, ^24^, ^25^, ^26^, ^27^, ^28^, ^29^, ^30^, ^31^, ^32^, ^33^, ^34^, ^35^, ^36^ separators,^37^, ^38^, ^39^, ^40^ and electrolytes^41^, ^42^, ^43^, ^44^, ^45^ to resolve these issues, some of which are rather dangerous, the results have been at best minimal in improving current Li‐S battery technology. As a result, the cycling life and Coulombic efficiency of Li‐S batteries, especially in the high‐rate regime, are still far behind the state of the art in lithium‐ion battery technology.

It is well known that the solid electrolyte interface (SEI), which is a manifestation of the decomposition of electrolytes and solutes, functions as the passivation layer and is an important arbiter in the lifespan of a battery.^46^ A high‐quality SEI can effectively protect the electrode while an inferior SEI gradually undermines the electrode performance. Although a unilateral SEI (in either the sulfur‐host cathode or lithium anode) was found effective in enhancing the performance of Li‐S batteries,^47^, ^48^ issues related to the dendrite growth and shuttle effect could not be resolved simultaneously.

**Figure 1:**
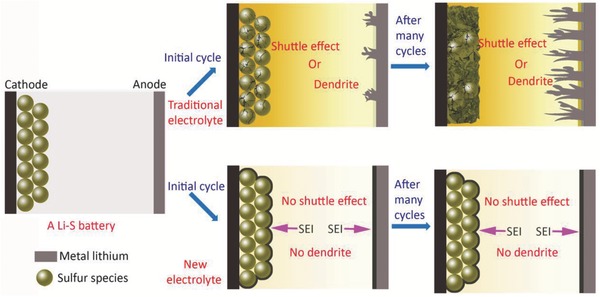
Conception of a bilateral solid electrolyte interface (SEI). With the new electrolytes described in this study, the battery forms a bilateral SEI on the sulfur‐host cathode and the lithium anode. Even after hundreds of cycles, the robust bilateral SEI effectively suppresses the dendrite growth on the lithium anode and the shuttle effect on the sulfur host cathode. On the other hand, with traditional electrolytes, the sulfur host cathode will deteriorate or severe dendrite growth will occur on the lithium anode during repeated cycling.

Here we report for the first time the development of new electrolytes for Li‐S batteries that promote the simultaneous formation of the bilateral SEIs on both electrodes. We find that the bilateral SEI forms readily during the initial cycle, which then effectively prevents the shuttle effect on the sulfur‐host cathode and dendrite growth on the metal lithium anode (**Figure** **1**). Compared to traditional electrolytes, the new electrolytes support ultralong cycling stability with a nearly ≈100% Coulombic efficiency owing to the effectiveness of the bilateral SEI in limiting polysulfide dissolution and dendrite growth. For example, when using sulfur‐doped polyacrylonitrile (SPAN) as the cathode, the Li‐S batteries exhibit a high utilization of sulfur (over 80% at 0.15 C, 1 C = 1675 mA h g^−1^|_sulfur_), superior rate performance, excellent Coulombic efficiency (≈100%), long cycling life (1000 cycles at 1 C and 2400 cycles at 7.5 C with capacity retention of 86.6% and 82.3%, respectively), outstanding ultrafast‐charge/slow‐discharge performance (charged within minutes and discharged for more than 10 h by lighting two light‐emitting diodes), and ultralow self‐discharge (cycled after storage for over 130 d with a capacity retention of 94.9%). More importantly, the industrial grade Li‐S pouch cells with 400 mA h capacity were constructed and cycled for 100 cycles with capacity retention of 67%.

## 2 Results and Discussion

**Figure 2:**
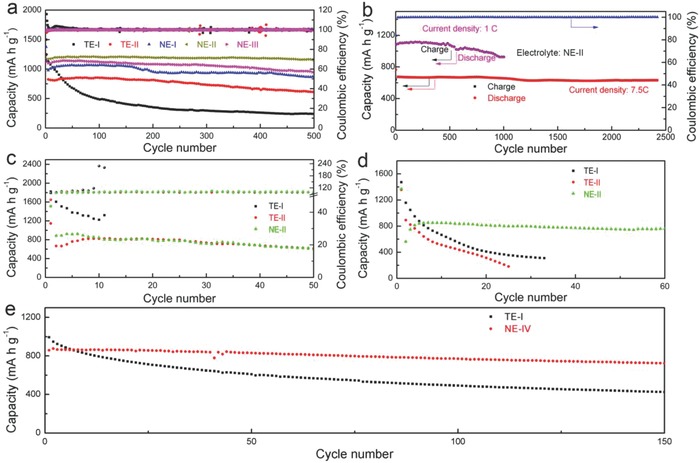
The electrochemical performance of new electrolytes in various cathode materials. a) Cycling stability of Li‐S batteries at 1.5 C, 42% sulfur content (SPAN‐42%). The batteries could deliver capacity of 250, 637, 909, 1218, and 985 mA h g^−1^ with TE‐I, TE‐II, NE‐I, NE‐II, and NE‐III electrolytes, corresponding to a capacity retention of 20.6%, 71.5%, 91.9%, 99.2%, and 84.5%, respectively. b) Long‐term cycling performance of Li‐S batteries (SPAN‐42%) with NE‐II at 1 C (1000 cycles) and 7.5 C (2400 cycles) with capacity retention of 86.6% and 82.3%, respectively. c) Cycling performance of Li‐S batteries with a porous carbon cathode with 40% sulfur content (PC@S) at 0. 5 C with TE‐I, TE‐II, and NE‐II electrolytes. d) Cycling stability of Li‐S batteries at 1.5 C, 62% sulfur content (SPAN‐62%) with TE‐I, TE‐II, and NE‐II electrolytes. e) Cycling performance of batteries at 0.5 C with Ketjen black cathode with 70% sulfur content (KB@S) and TE‐I and NE‐IV electrolytes.

In this study, over 60 compositions of electrolytes were prepared for purposes of investigating the electrochemical performance of Li‐S batteries. First, a SPAN was prepared and used as the active material due to its facile synthesis method and scalable production. The characterization and analysis of SPAN are shown in Figure S1 (Supporting Information) and Note S1 (Supporting Information). The cycling stability and corresponding Coulombic efficiency of batteries with different traditional electrolyte compositions at the current density of 7.5 C are shown in Figure S2 (Supporting Information). It is obvious that the cycling stability of the batteries with ethylene carbonate (EC) or propylene carbonate (PC) is much superior to that of either dimethyl carbonate (DMC) or ethyl methyl carbonate (EMC). When cycling for 500 cycles at 1.5 C (**Figure** **2**a), the batteries delivered a reversible capacity of 250, 637, 909, 1218, and 985 mA h g^−1^ with two traditional electrolytes (1 m lithium bis(trifluoromethanesulfonyl)imide (LiTFSI) in dimethyl ether (DME): dioxolane (DOL) = 1:1, volume ratio, referred as TE‐I for traditional electrolyte‐I; and 1 m LiPF_6_ in EC:DMC:EMC = 1:1:1, volume ratio, referred as TE‐II for traditional electrolyte‐II) and three new electrolyte (1 m Li(PF_6_)_0.3_(TFSI)_0.7_ in EC_0.1_:DMC_0.1_:EMC_0.1_:DME_0.35_:DOL_0.35_, volume ratio, referred as NE‐I; 1 m LiTFSI in EC_0.5_DME_0.25_DOL_0.25_, volume ratio, referred as NE‐II; and 1 m LiTFSI in PC_0.8_DME_0.1_DOL_0.1_, volume ratio, referred as NE‐III), which corresponds to capacity retentions of 20.6, 71.5, 91.9, 99.2, and 84.5%, respectively. The charge/discharge profiles after the second cycle (Figure S3, Supporting Information) also provide evidence for the shuttle effect with the TE‐I electrolyte. The rate performances of the batteries also suggest the superior electrochemical performance of Li‐S batteries with new electrolytes (Figure S4, Supporting Information). The kinetic analyses were performed to understand the different rate characteristics of the five electrolytes (the kinetic analyses derived from the CV curves are presented in Figure S5, Supporting Information), which revealed that the Li^+^ diffusion coefficient of the batteries with TE‐I, TE‐II, NE‐I, NE‐II, and NE‐III are 3.81 × 10^−9^, 1.75 × 10^−8^, 6.61 × 10^−9^, 2.49 × 10^−8^, and 1.02 × 10^−8^ cm^2^ s^−1^, respectively. The high Li^+^ diffusion coefficient of NE‐II and NE‐III is beneficial and consistent with its rate performance. Furthermore, the electrochemical impedance spectroscopy in Figure S6 (Supporting Information) suggests that the impedance of batteries after cycling is much lower compared to fresh batteries, and is largely due to the formation of a stable SEI. As evident in Figure 2b, even at a high current density of 7.5 C the battery with NE‐II electrolyte exhibited a favorable reversible capacity of 550 mA h g^−1^ after 2400 cycles, with superior capacity retention of 82.3% and a corresponding capacity fade of 0.0073%; and the battery delivered a reversible capacity of 940 mA h g^−1^ after 1000 cycles at 1 C with a capacity retention of 86.6%.

We also prepared a porous carbon @sulfur (PC@S, 40% sulfur content) electrode to investigate the effect of bilateral SEI (Figure 2c), and found that the Coulombic efficiency of the battery with TE‐I was inferior due to the shuttle effect. To confirm that the superior property of the Li‐S battery is not due to the relatively low concentration of S in the SPAN and PC@S, we prepared SPAN samples with a higher sulfur content of 62% (SPAN‐62%). The cycling properties of the SPAN‐62% in TE‐I, TE‐II, and NE‐II electrolytes are shown in Figure 2d, and the battery with NE‐II electrolyte exhibited a more stable cycle performance relative to that with TE‐I and TE‐II electrolytes. The enhanced performance of the battery with NE‐II is due to the formation of a bilateral SEI. In addition, a higher sulfur content cathode of Ketjen black @sulfur (KB@S, 70% sulfur content) was also prepared. We further found that by adding a 1% vinylene carbonate (VC) additive to TE‐I electrolyte (denoted as NE‐IV), the KB@S battery delivered more stable cycle performance relative to its performance in the absence of the VC additive (Figure 2e). The data in Figure 2 collectively demonstrated the significant enhancement of electrochemical performance with new electrolytes. The bilateral SEI could protect both electrodes during subsequent cycling, and mitigate the dendrite growth and shuttle effect to further enhance the electrochemical performance. For convenience, the SPAN‐42% was used in subsequent studies to further investigate the influence of the new electrolytes.

**Figure 3:**
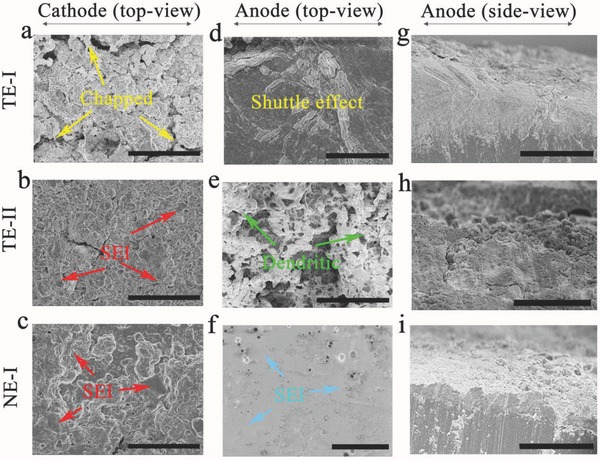
The morphology of electrodes after 150 cycles. a,d,g) Traditional electrolyte I (TE‐I). b,e,h) Traditional electrolyte II (TE‐II). c,f,i) New electrolyte I (NE‐I). Scale bars: a–f,h) 50 µm; g,i) 100 µm. a–c) Top view of the SPAN electrode which showed that it had roughened when TE‐I was used, while it remained intact with TE‐II and NE‐I due to the formation of SEI. d–f) Top view of the Li anode which showed the dendrite growth when TE‐II was used and the shuttle effect with TE‐I, while it became smooth with NE‐I on account of the SEI on the Li anode. g–i) Side view of the Li anode which showed that it remained ordered with TE‐I but crumbled with TE‐II, and became dense with NE‐I.

To confirm the formation of the bilateral SEI, scanning electron microscopy (SEM) images of the SPAN and Li electrodes in their pristine state and after a single cycle in TE‐I, TE‐II, and NE‐I electrolytes were analyzed (see Figure S7, Supporting Information). Unlike the pristine electrodes of SPAN and Li anode, in TE‐I the SPAN electrodes exhibited few morphological changes while the Li anode became relatively smoother, indicating that while the TE‐I barely reacted with the SPAN electrode, an SEI was formed on the Li anode. Conversely, an SEI was observed on the SPAN electrode in TE‐II and the surface of the Li anode appeared rough, which could be interpreted as the formation of dendrites on the Li anode. Notably, when the NE‐I electrolyte was used, both the SPAN and Li electrodes became smoother relative to their respective morphologies in TE‐I and TE‐II, implying the formation of the bilateral SEI on both electrodes. Interestingly, even after 150 cycles the SPAN electrode clearly revealed the absence of the SEI with the TE‐I electrolyte as evidenced by its roughened electrode surface (**Figure** **3**a). However, the SEI films that formed on the SPAN electrode in both the TE‐II (Figure 3b) and NE‐I (Figure 3c) electrolytes remained robust. For the Li anode with TE‐I (Figure 3d) or TE‐II (Figure 3e) electrolytes, both the shuttle effect and dendrite growth hindered batteries performance, while the Li surface coated with the NE‐I electrolyte remained smooth (Figure 3f) due to the formation of the bilateral SEI on both electrodes. The side view of the Li anode also provides respective evidence for the shuttle effect and the dendrite growth with the TE‐I (Figure 3g) and TE‐II (Figure 3h) electrolytes, respectively. The thin lithium deposition (in the side view of the Li anode with NE‐I) reveals that the bilateral SEI can effectively suppress the dendrite formation and shuttle effect (Figure 3i).

**Figure 4:**
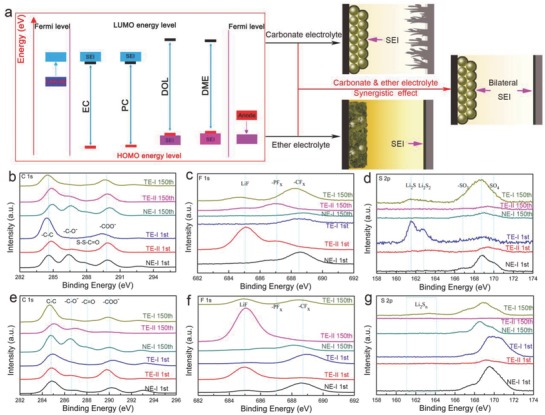
a) Schematic of bilateral solid electrolyte interface. b–g) XPS analyses of the surface of electrodes. b–d) SPAN electrode. e–g) Li anode.

The formation mechanism of unilateral SEI and bilateral SEI are consistent with density functional theory (DFT) calculations. The organic electrolytes used in Li‐S batteries were evaluated by comparing their highest occupied molecular orbital (HOMO) and the lowest unoccupied molecular orbital (LUMO) energy levels with respect to the Fermi energy levels of the electrodes. According to the molecular orbital theory, the electrophilic/nucleophilic substitution is favored at the orbitals where the HOMO/LUMO charge density is high. In particular, the nucleophilic (easy to accept electrons)/electrophilic (easy to donate electrons) substitution occurs in the electrolyte whenever its LUMO/HOMO energy level is lower/higher than the Fermi level in the electrode. A DFT (with a level of B3LYP/6‐31G) based calculation was used to investigate the electronic structures of both the ether (DOL) and carbonate solvents (EC, DMC, EMC, PC). We determined that the HOMO and LUMO energy levels of carbonate solvents were lower than the corresponding energy levels for ether solvents, indicating that these carbonate solvents were necessary for the nucleophilic reactions, while the ether solvents involved in the electrophilic reactions (Figure S8, Supporting Information). Therefore, in a typical Li‐S battery with carbonate‐based electrolytes, the SEI will form principally on the sulfur‐host cathode; and with ether‐based electrolytes the SEI will be formed on the lithium anode (**Figure** **4**a). Hence, to promote the simultaneous formation of a bilateral SEI on the sulfur‐host cathode and lithium anode, we combined the ether‐based solvents with carbonate‐based solvents in various proportions to identify an ideal electrolyte composition. With the new electrolyte compositions a bilateral SEI in an Li‐S battery can be readily formed during the initial cycle, which then effectively prevents the shuttle effect and dendrite growth, leading to an enhanced cycling stability. On the other hand, in Li‐S batteries that use traditional electrolytes, the absence of the bilateral SEI results in a serious battery deficiency caused by either the dendrite growth or the shuttle effect.

To verify the conjectured formation mechanism of the bilateral SEI, X‐ray photoelectron spectroscopy (XPS) was performed to quantify the SEI (TE‐I, TE‐II, and NE‐I as electrolytes) on SPAN and Li electrodes after a single cycle, and after 150 cycles. The high‐resolution C 1s, F 1s, and S 2p XPS spectra of the SPAN cathode after both a single cycle and 150 cycles in different electrolytes are shown in Figure 4b–d. The C 1s peak at ≈286.5 eV (—C—O^−^) is evident when the SPAN cathode was cycled in TE‐II and NE‐I electrolytes, signaling the formation of the SEI with the use of carbonate‐based electrolytes. With NE‐I, another peak located at ≈287.8 eV due to the formation of S—S—C=O bonds is evident, which is a result of the polysulfide reaction with the carbonate electrolyte.^49^ The single‐phase reaction in carbonate‐based solvent within the TE‐II prevented any obvious S—S—C=O bond formation, however. Furthermore, even after 150 cycles, the C 1s peaks remained nearly identical to that observed after the initial cycle, indicating a relative stability of the carbon bonds and providing further proof that the SEI was mainly formed during the initial cycle. Although the F 1s spectra (Figure 4c) imply the decomposition of –PF_6_ in TE‐II due to the formation of LiF, no such evidence was found with TE‐I and NE‐I.^50^ For the S 2p spectra (Figure 4d), however, no peaks at 160–165 eV were observed with TE‐II and NE‐I. Generally, the presence of these peaks signal the nucleophilic attack in carbonate electrolytes, which generates high valence sulfur species on the surface of the SPAN electrode. This scenario is consistent with that revealed by the C 1s spectra, and the reaction of polysulfides with carbonate electrolytes could further enhance the stability of SEI. A similar phenomenon characterizes the S 2p XPS of the SPAN electrode with both the NE‐II electrolyte and the KB@S cathode (Figure S9a,b, Supporting Information).

The high‐resolution C 1s, F 1s, and S 2p spectra in Figure 4e–g suggest the formation of the SEI in all electrolytes for the Li anode, which is confirmed by the C 1s spectra. Specially, the —C—O^−^ bond was enhanced after 150 cycles in NE‐I and TE‐I compared to that observed after a single cycle, which is attributed to the polymerization of the DOL. The F 1s spectra of the Li anode is similar to that of the SPAN cathode indicating a similar decomposition of both the –PF_6_ and the –TFSI during cycling in the SPAN and Li electrodes. The S 2p spectra of Li anode shows a broad peak of Li_2_S*_n_* indicating a possible migration of polysulfides to the Li anode to form Li_2_S*_n_*, and the presence of the RO‐Li (alkoxy lithium) peak in the Li 1s spectra (Figure S9c, Supporting Information) of the Li anode after a single cycle suggests that the Li anodes undergo similar reaction in TE‐I and NE‐I.^51^ After 150 cycles, the bond of RO‐Li in TE‐I and NE‐I electrolytes is weakened, but strengthened in TE‐II, a phenomenon attributed to the polymerization of DOL with RO‐Li in the TE‐I and NE‐I, consistent with the C 1s XPS. The bilateral SEI was observed to protect both electrodes during subsequent cycling, and mitigate the dendrite growth and shuttle effect which further enhanced the electrochemical performance. In contrast, only unilateral SEI formed on either the lithium anode or SPAN cathode with TE‐I or TE‐II, respectively, which resulted in a severe shuttle effect and/or dendrite growth with continuous cycling.

Most striking is the formation of the bilateral SEI during the initial discharge, as evident in the SEM images of both electrodes with the NE‐I electrolyte (Figure S10, Supporting Information). Furthermore, the ratio of initial charge capacity and the following discharge capacity with NE‐I was statistically ≈1 (Figure S11, Supporting Information), implying that the initial charge capacity is almost equal to the following discharge capacity with essentially no capacity loss (no SEI formation) during the initial charge process.

**Figure 5:**
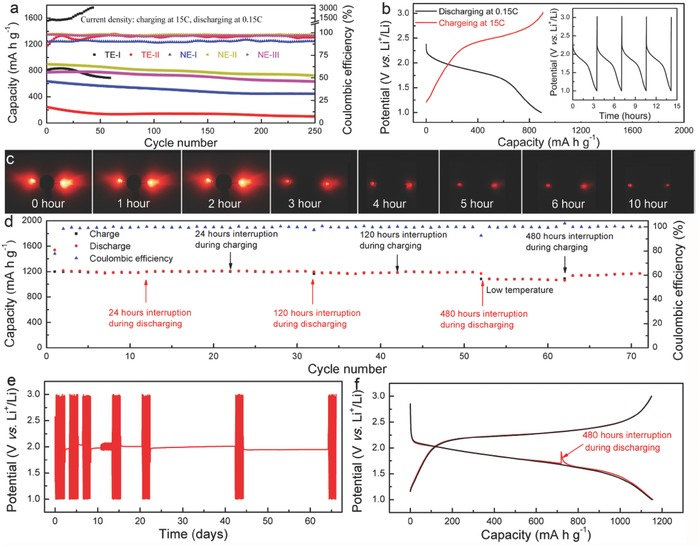
The electrochemical performance. a) Cycling performance of an Li‐S battery with a charging current of 15 C and a discharging current of 0.15 C. b) Typical discharge–charge profiles of an Li‐S battery with NE‐II at a charging current of 15 C and a discharging current of 0.15 C. Inset: charge and discharge cycles. The Li‐S battery with a bilateral SEI could be fully charged in minutes and discharged for over 3 h. c) The battery could be fully charged within minutes and power two LEDs for over 10 h. d–f) Charge/discharge storage properties of an Li‐S battery at 0.3 C. d) Long‐term cycling performance. e) The voltage–time profile of an Li‐S battery which was intermittently cycled with varying durations of storage for over 65 d showed no obvious deterioration. f) Typical discharge–charge profiles after storage for 480 h.

For practical applications in electric vehicles, continuous usage of a battery for a long period of time (slow discharge) and full charge in a very short time (fast charge) are of great importance. Consequently, we evaluated the ultrafast charge/slow discharge characteristics of batteries with TE‐I, TE‐II, NE‐I, NE‐II, and NE‐III electrolytes. The cycling performance depicted in **Figure** **5**a suggests a less than ideal configuration of batteries with TE‐I and TE‐II, as is evident from their poor Coulombic efficiency and low capacity, respectively. Remarkably, the NE‐I, NE‐II, and NE‐III electrolytes exhibited a superior performance due to their superior Li^+^ diffusion coefficient and the absence of the shuttle effect. After 250 cycles of charge at 15 C and discharge at 0.15 C, the batteries still exhibited superior capacity retention and excellent Coulombic efficiency. A typical charge/discharge profile of a battery with NE‐II showed rapid charging (within 2 min at 15 C) and long duration discharge (for over 3 h at 0.15 C) rates (Figure 5b and the inset). Lastly, these batteries could be fully charged within minutes and power two LED for over 10 h (Figure 5c).

A full cell was also assembled using lithiated graphite as the anode and the SPAN as the cathode and using the five electrolytes discussed in this study. As shown in Figure S12a (Supporting Information), during discharge the Li^+^ in graphite is released and transferred to the SPAN electrode leading to Li_2_S formation, a process which is reversed during charging. The potential plateaus of Li‐S and the Li‐graphite half‐cells are ≈2.0 and ≈0.1 V, respectively (Figure S12b, Supporting Information) while that of the S‐graphite full cell is ≈1.85 V. Although the cycling performance (Figure S12c, Supporting Information) of the full cells using different electrolytes at 0.75 C indicated a rapid decline in capacity with the use of TE‐I and TE‐II, the capacity remained robust with the NE‐I, NE‐II, and NE‐III electrolytes. For repeated discharge/charge cycles of more than 200 cycles at a current density of 0.75 C, the full cells using new electrolytes delivered a remarkable high reversible capacity over 1000 mA h g^−1^ with a capacity retention exceeding 80% (based on the second discharge). The typical discharge/charge profiles with NE‐I illustrated in Figure S13 (Supporting Information). Moreover, the Coulombic efficiency could reach ≈100% after several cycles. A review of the literature indicates that this cycling stability of the full S‐graphite has thus far yielded the most superior performance. These findings and the kinetic analyses of the S‐graphite full cell (Figure S14 and Note S2, Supporting Information) clearly indicate the great potential of these S‐graphite (lithiated) full cells.

Although the new NE‐I, NE‐II, and NE‐III electrolytes exhibit superior electrochemical performance compared with their traditional TE‐I and TE‐II counterparts due to the formation of bilateral SEI (Figures 2 and 3; Figures S7 and S15, Supporting Information), NE‐II was found superior to the other two given the higher Li^+^ diffusion coefficient, discharge capacity, and capacity retention. Therefore, a detailed study of the electrochemical performance of NE‐II was undertaken to determine the reasons for this superiority. First, various mass loadings (ranging from 1.8 to 7.7 mg cm^−2^) were created by controlling the thickness of the electrode materials. As shown in Figure S16 (Supporting Information), the cycling stability of batteries with different mass loadings at the current density of 0.75 C with NE‐II clearly indicated a capacity that remained stable for over 200 cycles with a Coulombic efficiency of nearly 100%. Further, when the electrode is composed of 90% active materials and a 10% binder with a mass loading of 10–15 mg cm^−2^, the battery still exhibited excellent electrochemical performance. A high‐capacity electrode over 1000 mA h g^−1^ at 0.15 C after 100 cycles is illustrated in Figure S17 (Supporting Information), which retains this quality even with electrodes subjected to pressures of 0 and 9 MPa. Given the critical importance of the electrolyte‐to‐sulfur ratio (E/S) for the practical application of Li‐S batteries, we investigated the effect of different E/S ratios during cycling.^52^ We observed a slight difference in performance with a change in E/S ratio from 6:1 to 15:1 (Figure S18, Supporting Information), indicating that the E/S is not the limiting factor as long as the electrolyte could fully infiltrate the electrode.

As an additional test for the robustness of the Li‐S batteries discussed in this study, we evaluated their performance over a low temperature range from room temperature (≈25 °C) to ≈4 °C (Figure S19, Supporting Information). Though the capacity of Li‐S battery decreased slightly at low temperatures, it could be recovered when the temperature returned to the room temperature, signifying its superior adaptability to a change in temperature. The typical charge/discharge profiles of the Li‐S battery at room temperature and 4 °C is illustrated in Figure S20 (Supporting Information).

A secondary criterion for realistic application of Li‐S batteries is their self‐discharge properties. As shown in Figure 5d, an Li‐S battery with NE‐II electrolyte was charged and discharged at 0.3 C for over 70 cycles. We interrupted the discharge process at the 12th cycle, disconnected the battery for 24 h before continuing with the discharging process (indicated by the up‐pointing red arrow in Figure 5d). A similar 24 h long interruption was also implemented during the 22nd charge cycle as indicated by the down‐pointing black arrow in Figure 5d. We observed no reduction/increase of the total discharge/charge capacities at the 12th/22nd cycles, indicating nearly zero self‐discharge of the battery during the 24 h interruption. Similar measurements were performed by disconnecting the battery for 120 and 480 h during the discharging or charging process, with no significant change in the total discharge/charge capacities observed (Figure 5d). In addition to interrupting the discharge of the Li‐S battery at the 52nd cycle for 480 h (Figure 5d), a decrease in temperature to 4 °C, followed by a return to room temperature was observed during the 63rd charge cycle. Further, when cycled and stored over a 65 d period (Figure 5e), the battery still delivered a reversible capacity of 1169 mA h g^−1^, which corresponds to a capacity retention of 96.2% based on the capacity of second discharge. As indicated by the red discharge curve in Figure 5f, no change was observed in capacity after a 480 h interruption of the discharging process, indicating an excellent storage capability due to the mitigation of the polysulfide shuttle.

A further cycling and storage of the battery for an additional 100 cycles (Figure S21a, Supporting Information) only resulted in a marginal degree of degradation, with a reversible capacity of 1153 mA h g^−1^, and a capacity retention rate of 94.9% based on the capacity of the second discharge. At this point, the batteries were already cycled with a charge–discharge interruption exceeding 130 d (Figure S21b, Supporting Information); such an excellent charge storage performance of Li‐S batteries is crucial for their practical application.

**Figure 6:**
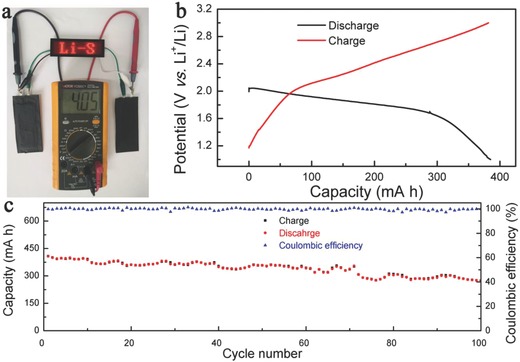
Industrial grade Li‐S pouch cells with capacity around 400 mA h. a) Photograph of the LED array which reads “Li‐S.” The LED array was powered by two Li‐S pouch cells. b) Typical discharge–charge profiles. c) Cycling stability for 100 cycles.

To further investigate the possibility of the Li‐S battery with a bilateral SEI for commercial applications, industrial grade Li‐S pouch cells based on NE‐II with a capacity of ≈400 mA h were fabricated. The optical photograph of two tandem Li‐S pouch cells connected in series yielded an output voltage of 4.05 V that could power the light‐emitting diode (LED) display (**Figure** **6**a). The typical charge/discharge profiles of the Li‐S pouch cells are depicted in Figure 6b. The Li‐S pouch cells could deliver a reversible capacity of 407 mA h and maintain a reversible capacity of 342 mA h after 60 cycles, with a capacity retention of 84% (Figure 6c). Notably, the Li‐S pouch cells still retained a capacity of 67% for a further 100 cycles.

## 3 Conclusion

Through the use of new electrolytes we conceptualized and demonstrated the formation of a bilateral SEI which resulted in high‐performance Li‐S batteries for practical applications. The bilateral SEI simultaneously suppressed the growth of dendrites on the Li anode and the deterioration of the sulfur‐host cathode, leading to outstanding electrochemical performance. Furthermore, the Li‐S batteries exhibited high utilization of sulfur, superior rate performance, long cycle stability with ≈100% Coulombic efficiency, and high performance of ultrafast‐charge/slow‐discharge, ultralow self‐discharge. The industrial grade high‐capacity Li‐S pouch cells also exhibited a stability of over a lengthy cycle, indicating methods to develop long lived Li‐S batteries with high energy and high power densities.

## Conflict of Interest

The authors declare no conflict of interest.

## Supporting information

Supplementary
