# Supplementary material for: Simultaneous Suppression of the Dendrite Formation and Shuttle Effect in a Lithium–Sulfur Battery by Bilateral Solid Electrolyte Interface
Source: Adv Sci (Weinh). 2018 Jul 23;5(9):1700934. doi: 10.1002/advs.201700934 (PMC6145423; doi:10.1002/advs.201700934)
Supplement: Supplementary file 1 — Supplementary [file ADVS-5-1700934-s001.pdf]

Copyright WILEY-VCH Verlag GmbH & Co. KGaA, 69469 Weinheim, Germany, 2018.

ADVANCED SCIENCE
Open Access

Supporting Information

for *Adv. Sci.,* DOI: 10.1002/advs.201700934

Simultaneous Suppression of the Dendrite Formation and Shuttle Effect in a Lithium–Sulfur Battery by Bilateral Solid Electrolyte Interface

Ling Fan, Suhua Chen, Jingyi Zhu, Ruifang Ma, Shuping Li, Ramakrishna Podila, Apparao M. Rao, Gongzheng Yang, Chengxin Wang,* Qian Liu, Zhi Xu, Lixia Yuan, Yunhui Huang, and Bingan Lu**

Copyright WILEY-VCH Verlag GmbH & Co. KGaA, 69469 Weinheim, Germany, 2016.

Supporting Information

Simultaneous Suppression of the Dendrite Formation and Shuttle Effect in a Lithium-Sulfur Battery by Bilateral Solid Electrolyte Interface

Ling Fan, Suhua Chen, Jingyi Zhu, Ruifang Ma, Shuping Li, Ramakrishna Podila, Apparao M. Rao, Gongzheng Yang, Chengxin Wang,* Qian Liu, Zhi Xu, Lixia Yuan, Yunhui Huang, Bingan Lu**

Dr. L. Fan, Dr. S. Chen, Dr. R. Ma, Dr. Q. Liu, Prof. B. Lu
School of Physics and Electronics, Hunan University, Changsha 410082, China
Dr. J. Zhu, Prof. R. Podila, Prof. A. M. Rao
Department of Physics and Astronomy, Clemson Nanomaterials Institute, Clemson University, Clemson, South Carolina 29634, United States
Dr. J. Zhu
Department of Mechanical and Aerospace Engineering, New York University, Brooklyn, New York 11201, United States
Dr. S. Li, Prof. L. Yuan, Prof. Y. Huang
Key Laboratory for Advanced Battery Materials and System (MOE), School of materials Science and Engineering, Huazhong University of Science and Technology, Wuhan, Hubei 43004, China
Prof. G. Yang, Prof. C. Wang
School of Materials Science and Engineering, Sun Yat-sen University, Guangzhou, 510275, China
Prof. Z. Xu, Prof. B. Lu
2D Material Technology Company Limited, Wing Lok Street, Sheung Wan, Hong Kong 999077, China
Prof. Z. Xu, Prof. B. Lu
Fujian Strait Research Institute of Industrial Graphene Technologies, Jinjiang, Fujian, 362200, China
*Corresponding to: luba2012@hnu.edu.cn (B. L), arao@g.clemson.edu (A. M. Rao), wchengx@mail.sysu.edu.cn (C. W)

Experimental Section

Materials and Methods

Chemicals

The lithium bis(trifluoromethane sulfonimide) (LiTFSI), lithium hexafluorophosphate ($LiPF_6$), ethylene carbonate (EC), dimethyl carbonate (DMC), ethyl methyl carbonate (EMC), propylene carbonate (PC), vinylene carbonate (VC), dimethyl ether (DME), dioxolane (DOL) were purchased from BASF. The polyacrylonitrile (PAN) was purchased from Aladdin, and all chemicals were used as received.

Preparation of electrolyte

For the purpose of investigating the effect of different electrolytes, two typical electrolytes of 1M LiTFSI in DME: DOL=1:1, (*v*/*v*, referred as TE-I, short for traditional electrolyte-I) and 1M $LiPF_6$ in EC: DMC: EMC=1:1:1 (*v*/*v*/*v*, refereed as TE-II, short for traditional electrolyte-II) were prepared. Furthermore, electrolytes with different volume ratios (1:9, 2:8, 3:7, 4:6 5:5, 6:4, 7:3, 8:2, 9:1) of TE-I and TE-II were also prepared, and these compositions were referred by the proportion of solvent in the electrolyte ($TE\text{-}I_xTE\text{-}II_{1\text{-}x}$, $0.0 \leq x \leq 1.0$). For the purpose of investigating the influence of the solute of LiTFSI and $LiPF_6$, different ratios of LiTFSI and $LiPF_6$ in DME: DOL=1:1 (*v*/*v*) were prepared, referred as $Li(PF_6)_x(TFSI)_{1\text{-}x}$ ($0.0 \leq x \leq 1.0$). Furthermore, 1M LiTFSI in different volume ratios of EC, DMC, and EMC with DME: DOL=1:1 (*v*/*v*) were also prepared, referred by the proportion of solvent in the electrolyte ($EC_xTE\text{-}I_{1\text{-}x}$, $DMC_xTE\text{-}I_{1\text{-}x}$, $EMC_xTE\text{-}I_{1\text{-}x}$, $0.0 \leq x \leq 1.0$). Another new electrolyte with 1% VC additive in 1M LiTFSI in DOL:DME (1:1, *v*/*v*) was prepared, and referred as NE-IV.

Synthesis of the cathode materials

The SPAN was synthesized by pyrolyzing PAN and sulfur powder. A homogeneous mixture of 2.5 g PAN (M_w=150,000) and 7.5 g of sublimed sulfur was created and heated to 450 °C

(with a heating rate of 5 °C min^{-1} under nitrogen atmosphere) and held at this temperature for 6 hours. The black colored product, referred as SP-42%, was used as the active material. A high sulfur content SPAN (SP-62%) was also synthesized using the method above but with a higher ratio S (PAN:S = 1:9) and a hold temperature of 300 °C. Ketjen black @sulfur (KB@S) was prepared with ratio of KB:S as 3:7 by first holding the temperature at 155 °C for 12 hours, and then raising it to 200 °C and holding for another 2 hours. Porous carbon @sulfur (PC@S) was prepared by hydrothermal-annealing glucose, then activation with KOH at 700°C, finally the sulfur was infiltrated with 40% content.

Material Characterization

Scanning electron microscope (SEM) and transmission electron microscopy (TEM) was used to characterize the microstructure, and XRD with a 2θ range of 5-80 using Cu Kα radiation (0.154056 nm) was used to characterize the crystal structure. An ESCALAB 250Xi was used for the XPS analysis, a Renishaw 2000 system was used for collecting the Raman spectrum, the WQF-510A was used to measure the FTIR transmission. Finally, a STA 449C thermoanalyzer with heating rate of 5 °C min^{-1} was used for the TGA analysis.

Electrochemical Characterization

Generally, the electrodes were prepared by coating the SPAN: carbon black: CMC (70:15:15, *w*/*w*/*w*) or SPAN: CMC (90:10, *w/w*) onto the current collector. For the assembly of the 2032 coin cells, Li foils were used as the counter electrode and the reference electrodes and the galvanostatic charge/discharge was measured using a voltage range of 1.0-3.0 V for all half cells. For the assembly of the full cells, a graphite powder: carbon black: CMC (8:1:1, *w*/*w*/*w*) mixture was coated onto the current collect and used as the anode (graphite anode), the SPAN electrode was used as the cathode, and the new electrolytes were used as the electrolyte. Prior to the assembly of the full cells, the graphite anodes were placed in direct contact with the Li foils for at least 1 hour to ensure lithiation of the graphite anodes. The galvanostatic charge/discharge measurements of the full cells were investigated over the voltage range of

0.8-3.0 V. All capacity calculations were based on the weight of sulfur. Cyclic voltammetry (CV) measurements were performed on an electrochemical workstation at various scan rates with a voltage range of 3.0-1.0 V for half cells and 3.0-0.8 V for full cells. The electrochemical impedance spectrum (EIS) measurements were conducted on an electrochemical workstation in the frequency range of 100 kHz~0.01 Hz.

As for the baseline data, the areal mass loading was approximately 1 mg cm^{-2} with the electrolyte amount encompassing the excess.

For the KB@S electrodes, the electrodes were prepared by coating the KB@S: carbon black: PVDF (85:5:10, *w*/*w*/*w*) onto the current collector, thus increasing the sulfur content in the entirety of the electrode to 60%, including the cathode materials, and the conductive additive and binder. The electrolyte used for KB@S is TE-I and NE-IV, 5 cycles with a voltage range of 0.3-1.0 V were performed to catalyze the formation of SEI in the sulfur cathode. The galvanostatic charge/discharge measurements were then performed with a voltage range of 1.3-2.8 V.

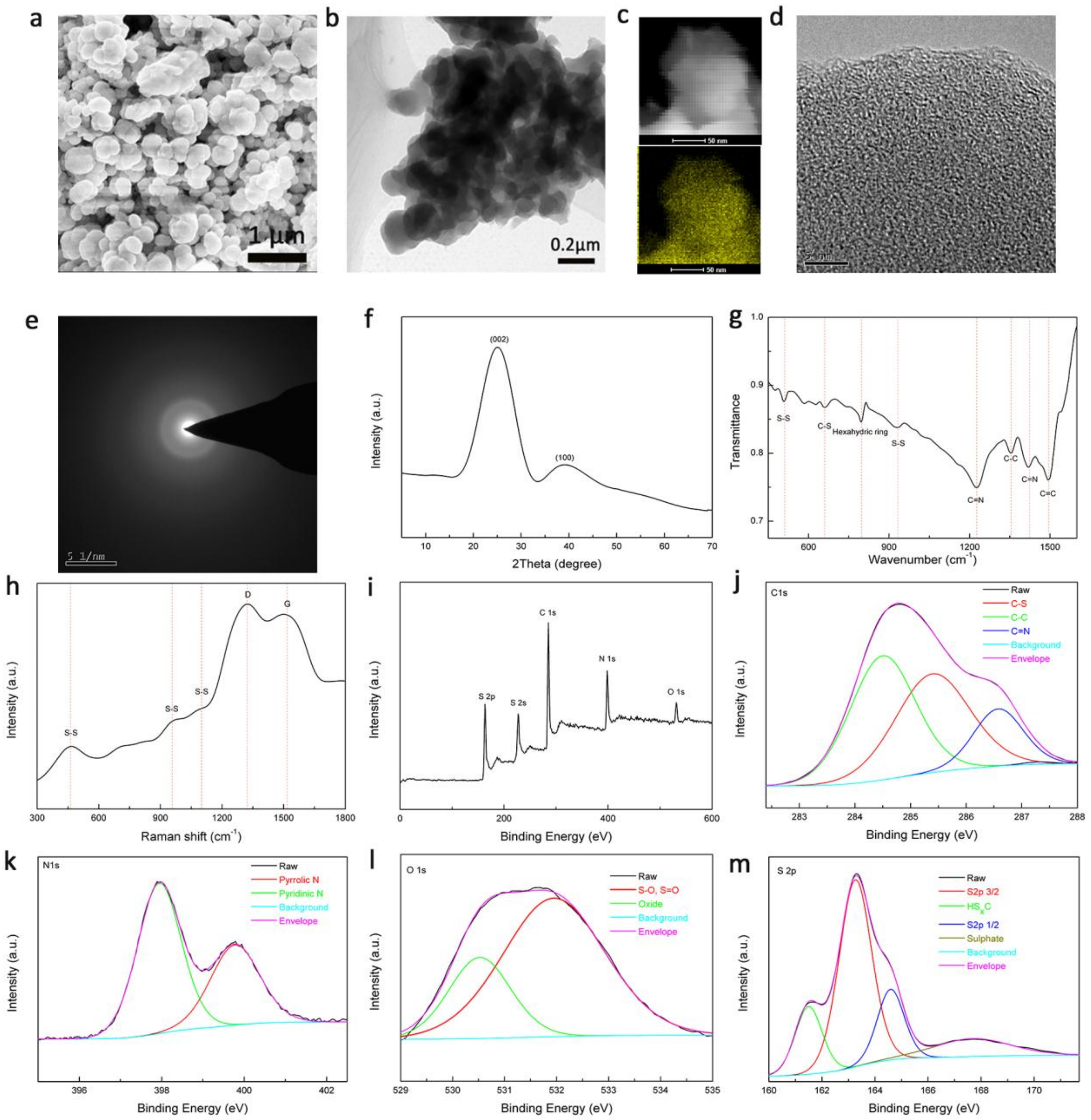

Figure S1. The morphology and structural characterization of SPAN. a, SEM image; **b**, TEM image; **c**, mapping of elemental sulfur; **d**, HRTEM image (scale bar, 5 nm); **e**, the SAED image; **f**, XRD pattern; **g**, FTIR transmission spectrum; **h**, Raman spectrum; **i**, full XPS survey; **j** C1s XPS profile; **k**, N1s XPS profile; **l**, O1s XPS profile; and **m**, S2p XPS profile.

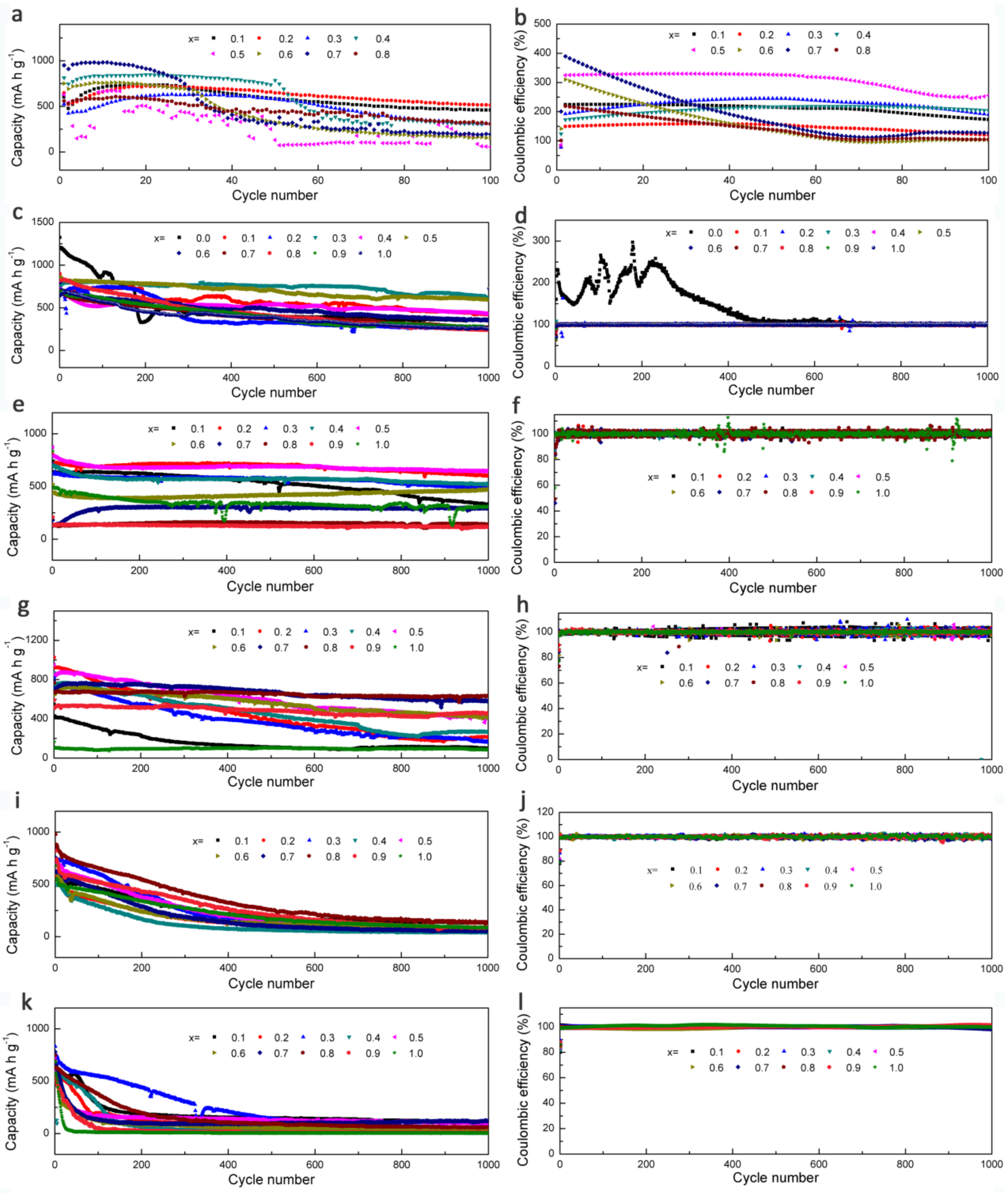

Figure S2. The cycling stability and Coulombic efficiency of batteries with different traditional electrolyte compositions at the current density of 7.5 C. a, b, $Li(PF_6)_x(TFSI)_{1-x}$ ($0.1 \le x \le 0.8$); **c, d,** TE-I_{1-x}TE-II_x ($0.0 \le x \le 1.0$); **e, f,** EC_xTE-I_{1-x} ($0.1 \le x \le 1.0$); **g, h,** PC_xTE-I_{1-x} ($0.1 \le x \le 1.0$); **i, j,** DMC_xTE-I_{1-x} ($0.1 \le x \le 1.0$); and **k, l,** EMC_xTE-I_{1-x} ($0.1 \le x \le 1.0$). Here, TE-I (1M LiTFSI in DME:DOL=1:1, volume ratio) and TE-II (1M $LiPF_6$ in EC:DMC:EMC=1:1:1, volume ratio) represent traditional electrolytes. The EC, PC, DMC and EMC correspond to ethylene carbonate, propylene carbonate, dimethyl carbonate and ethyl methyl carbonate, respectively.

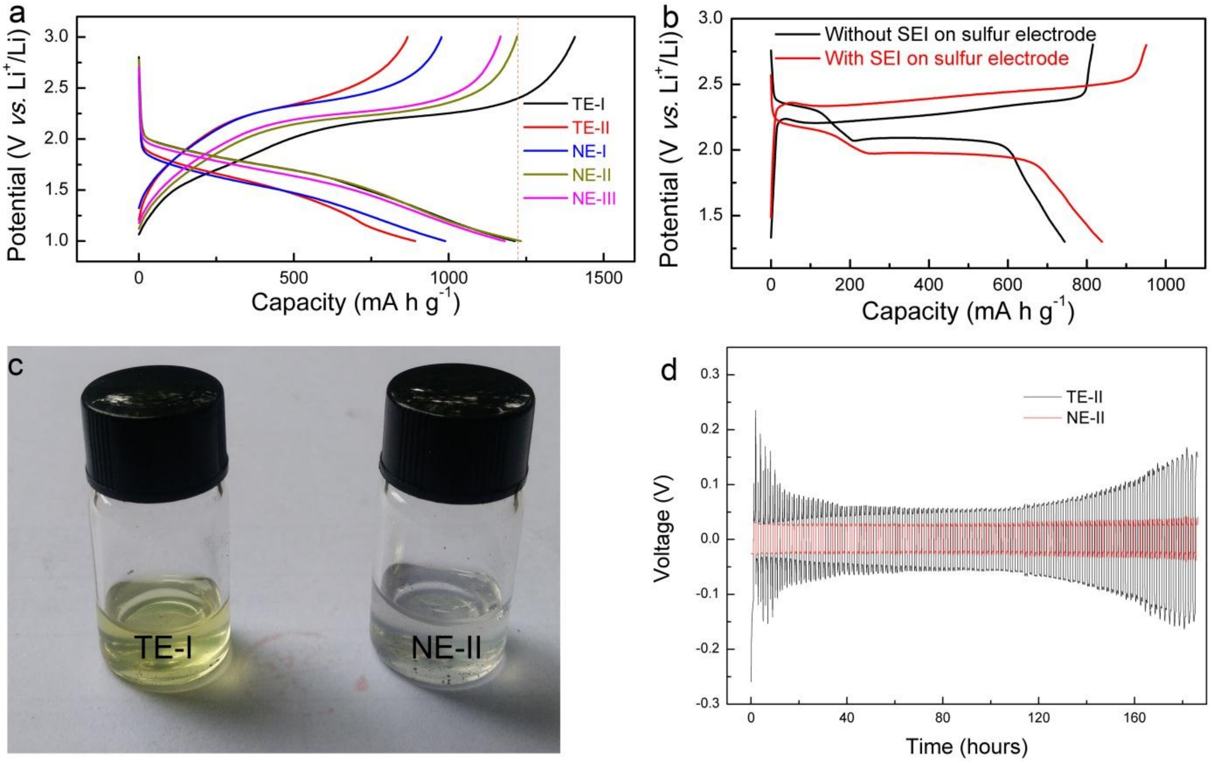

Figure S3. The second charge/discharge profiles at a current density of 1.5 C with different electrolytes. a, Li-S batteries (SPAN-42%); **b,** Li-S batteries (Ketjen black @sulfur electrode in NE-IV. **c,** The battery were disassembled and immersed in corresponding electrolyte. The colorless electrolyte demonstrating that no shuttle effect was appeared when using NE-II electrolyte. **d,** Electrochemical performance of symmetric Li||Li cells with NE-II and TE-II electrolyte. Capacity: 1 mA h cm^{-2}, current density:1 mA cm^{-2}. The low initial stripping/plating overpotential of NE-II electrolyte indicates the stripping/plating of Li with NE-II electrolyte is better than that with TE-II electrolyte.

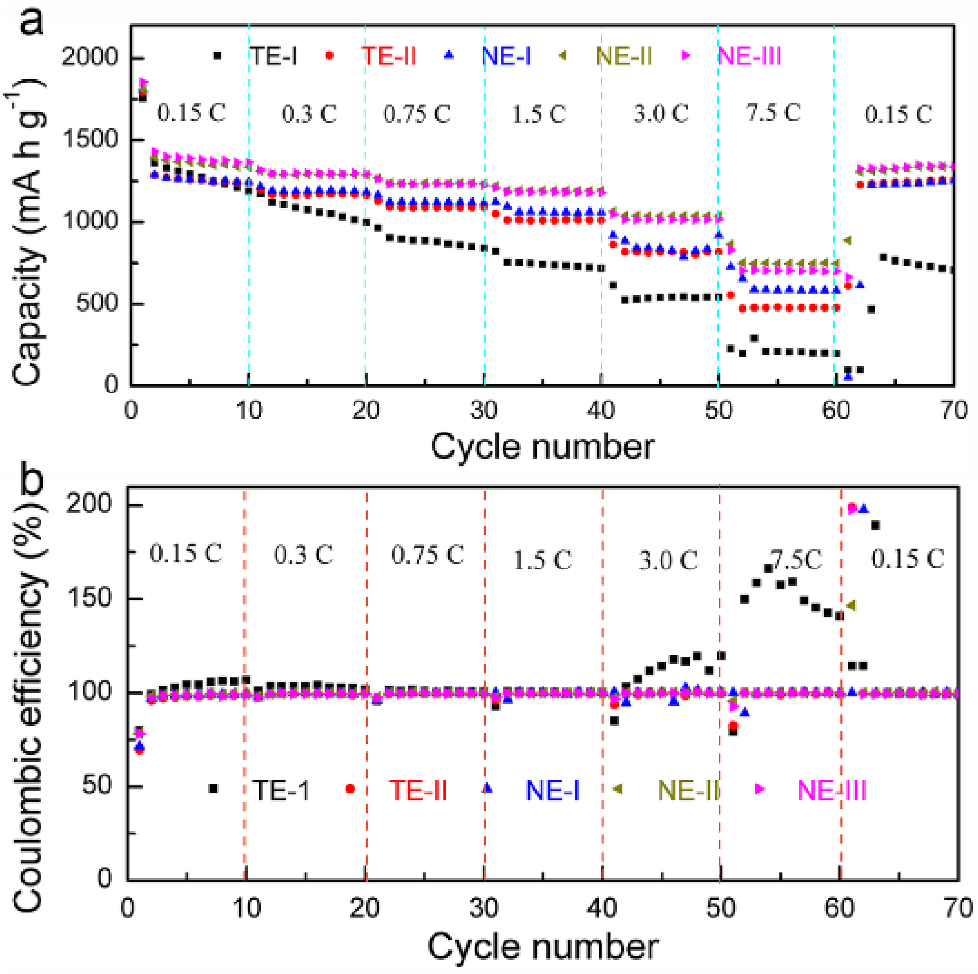

Figure S4. The Coulombic efficiency of Li-S batteries with the five electrolytes used in this study at various current densities. a, The rate performances of the batteries with all five electrolytes suggest that the capacity of the battery with TE-I fades rapidly; and **b,** the Coulombic efficiency at high rates are suppressed. While the battery capacity with TE-II was particularly low at high current densities, it was relatively high with the new NE-I, NE-II, and NE-III electrolytes and exhibited excellent Coulombic efficiency due to the formation of bilateral SEI. The corresponding rate capacities are summarized in Table S1.

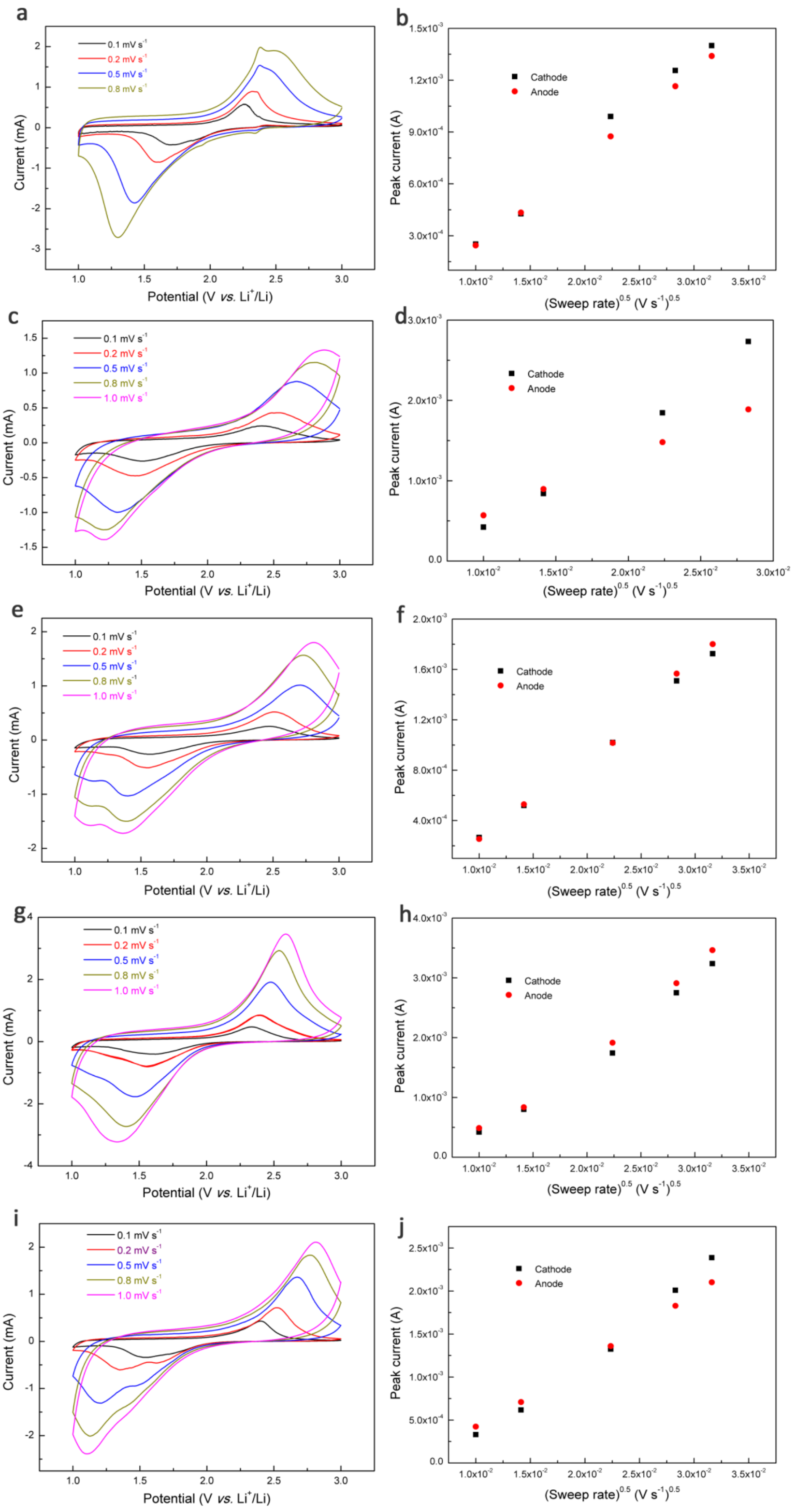

Figure S5. The kinetics analyses of a Li-S battery with the five different electrolytes described in this study. a, b, TE-I. **c, d,** TE-II. **e, f,** NE-I. **g, h,** NE-II. **i, j,** NE-III.

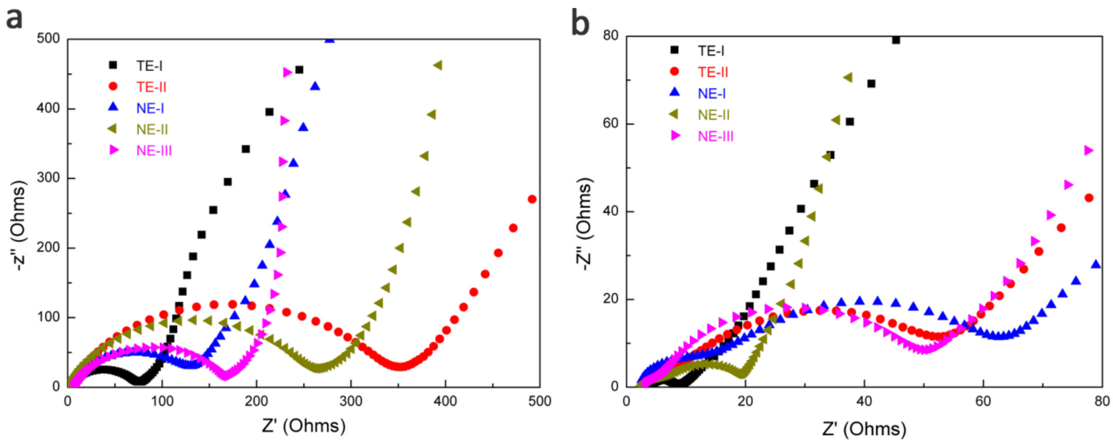

Figure S6. EIS of Li-S batteries before and after 150 cycles. a, Before cycling. **b,** After 150 cycles.

、

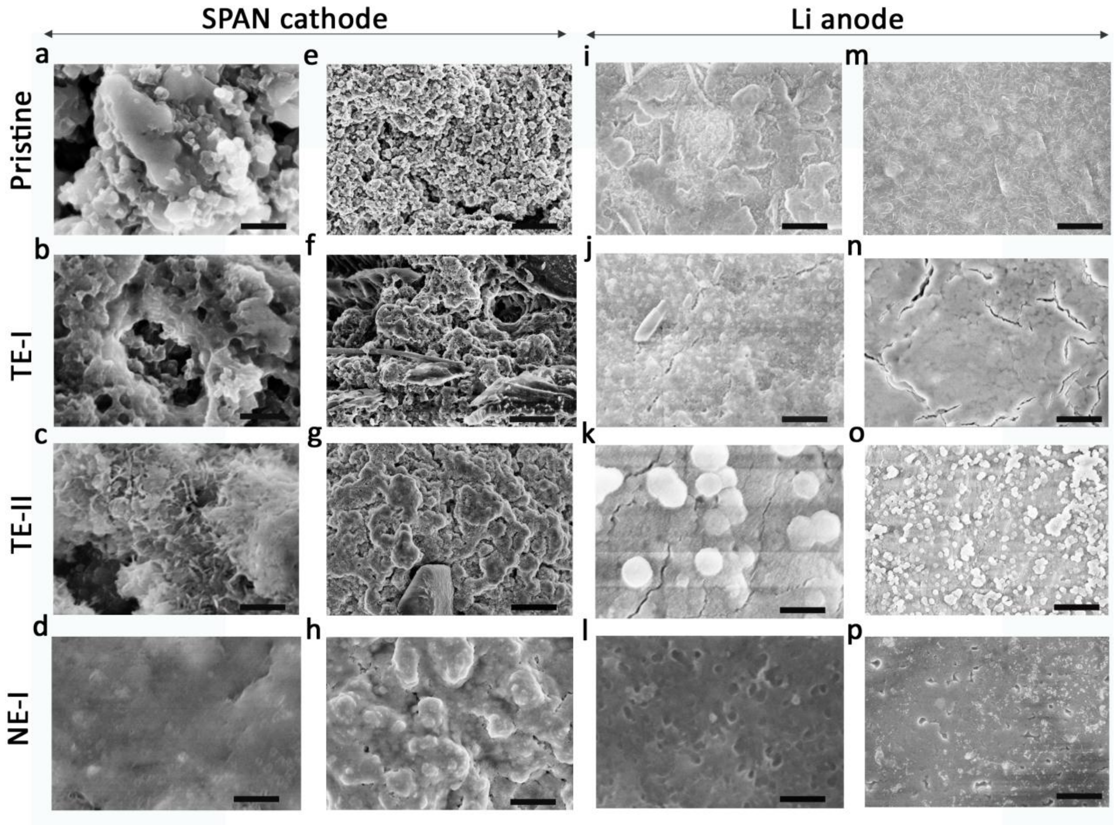

Figure S7. The SEM images of SPAN and Li electrodes before and after one cycle with different electrolytes. a-h, SPAN electrode. **i-p,** Li electrode. Images **a, e, i, m** correspond to pristine electrodes while images **b, f, j, n** correspond to electrodes after 1 cycle in TE-I. Likewise, images **c, g, k, o** correspond to electrodes after 1 cycle in TE-II, and images **d, h, l, p** correspond to electrodes after 1 cycle in NE-I. Scale bars: **a-d,** 0.5 μm; **e-h,** 5 μm; **i-l,** 0.5 μm; **j-l,** 5 μm.

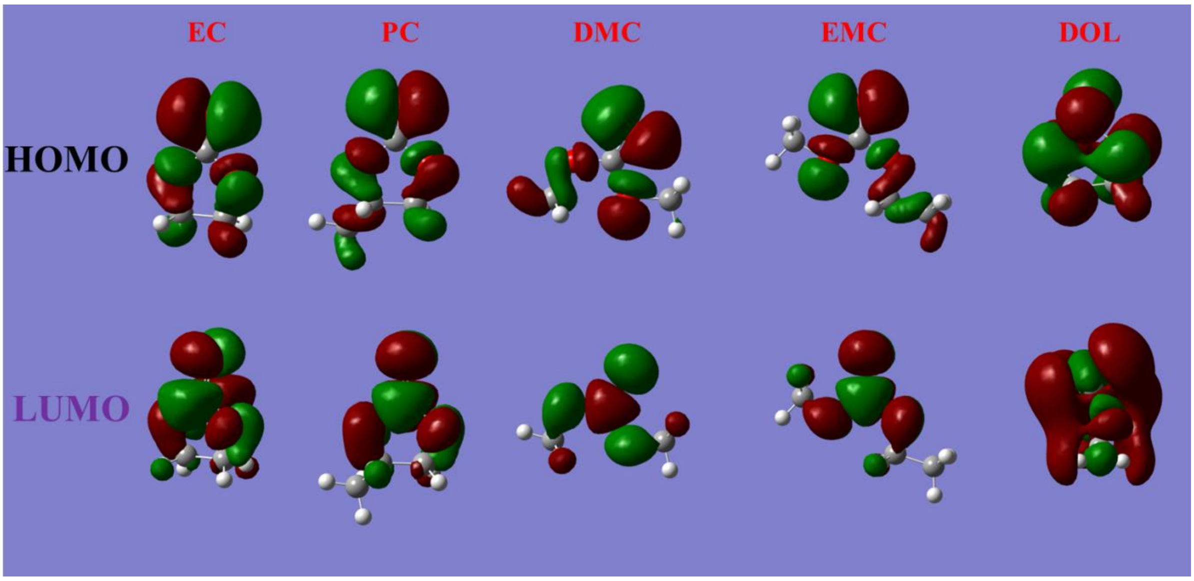

Figure S8. Highest occupied and lowest unoccupied molecular orbitals The highest occupied (HOMO) and lowest unoccupied (LUMO) molecular orbitals (top panel) and the corresponding energy levels (bottom panel) of typical solvents used in a Li-S battery: ethylene carbonate, EC; propylene carbonate, PC; dimethyl carbonate, DMC; ethyl methyl carbonate, EMC; dioxolane, DOL.

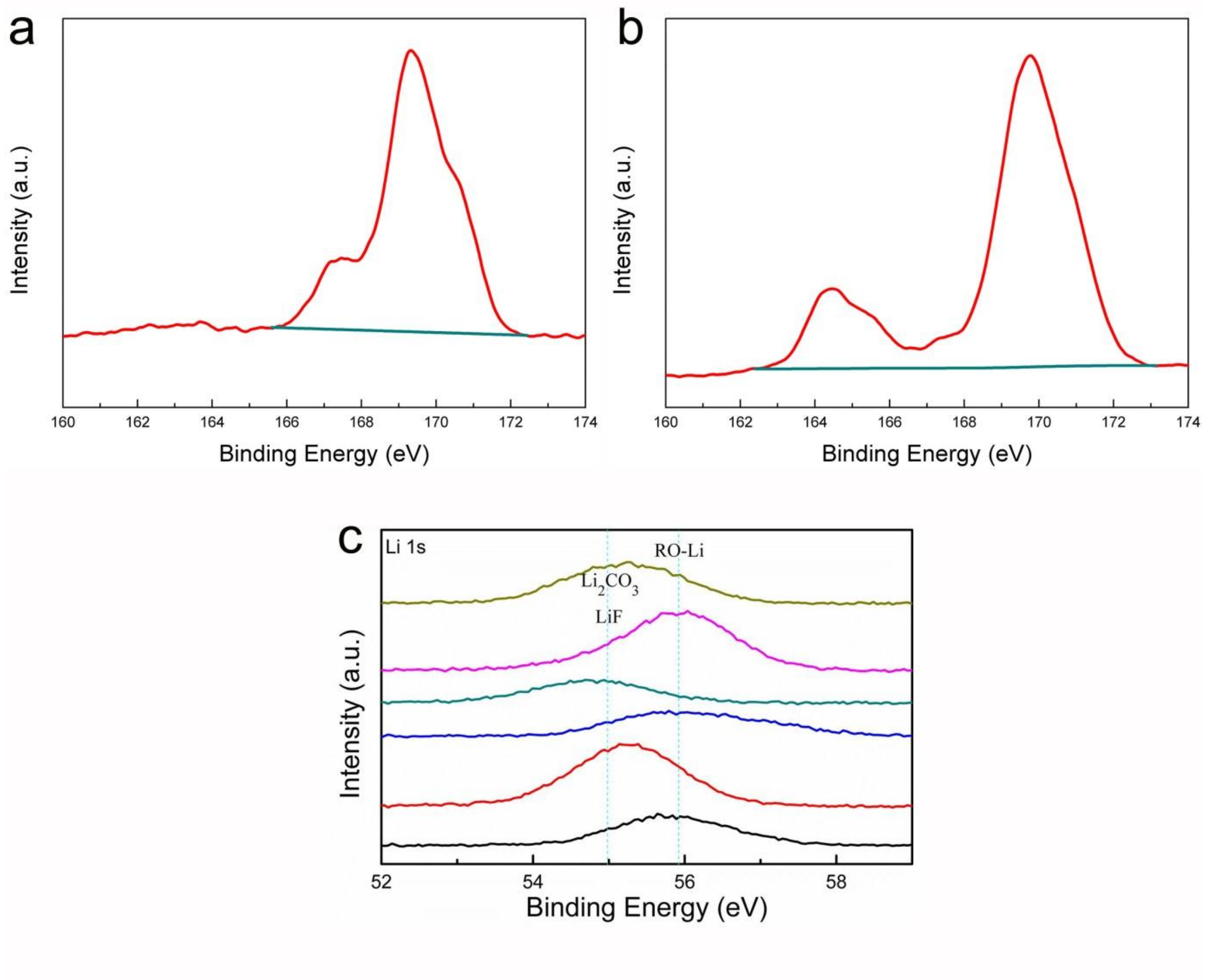

Figure S9. XPS analyses of the electrodes used in this study. **a,** The S2p XPS of the SPAN electrode with NE-II electrolyte after one cycle. **b,** The S2p XPS of the KB@S electrode with SEI formation. **c,** The Li1s XPS of the Li anode after one cycle and 150 cycles.

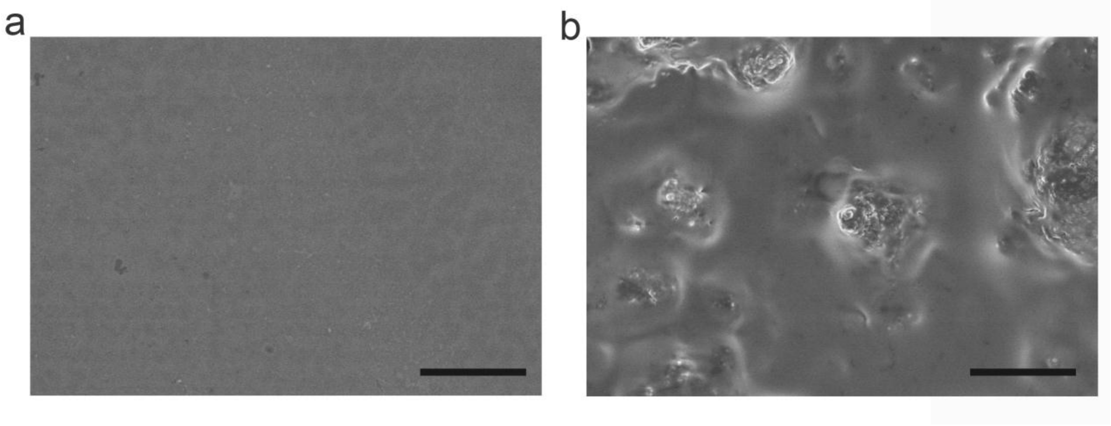

Figure S10. The SEM images of both electrodes after the initial discharge with NE-I electrolyte. a, Li anode. **b,** SPAN cathode. Scale bars: 20 μm.

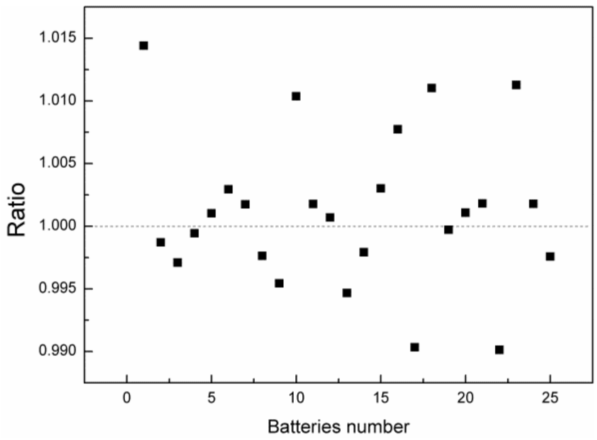

Figure S11. A statistical analysis of the ratio of the initial charge capacity to the following discharge capacity with NE-I.

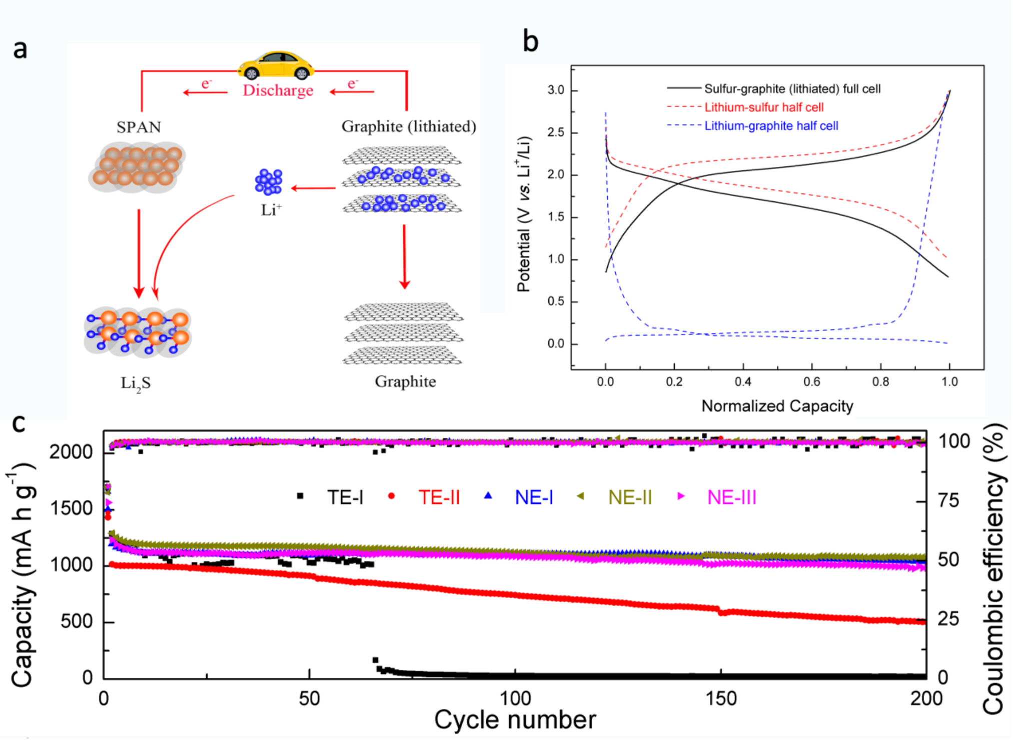

Figure S12. The electrochemical performance of sulfur-graphite (lithiated) full cells. a, Schematic diagram of a sulfur-graphite battery during the discharge process. Li^+ ions de-intercalate from the lithiated graphite anode during discharge and form Li_2S on the SPAN cathode. **b**, The normalized discharge/charge profiles of a sulfur-graphite full-cell, sulfur and graphite half-cell. The lithium-sulfur and lithium-graphite half-cell plateaus are approximately 2.0 V and 0.1 V, respectively, while that for the sulfur-graphite full cell is approximately 1.85 V. **c**, The cycling stability of the sulfur-graphite (lithiated) batteries with different electrolytes.

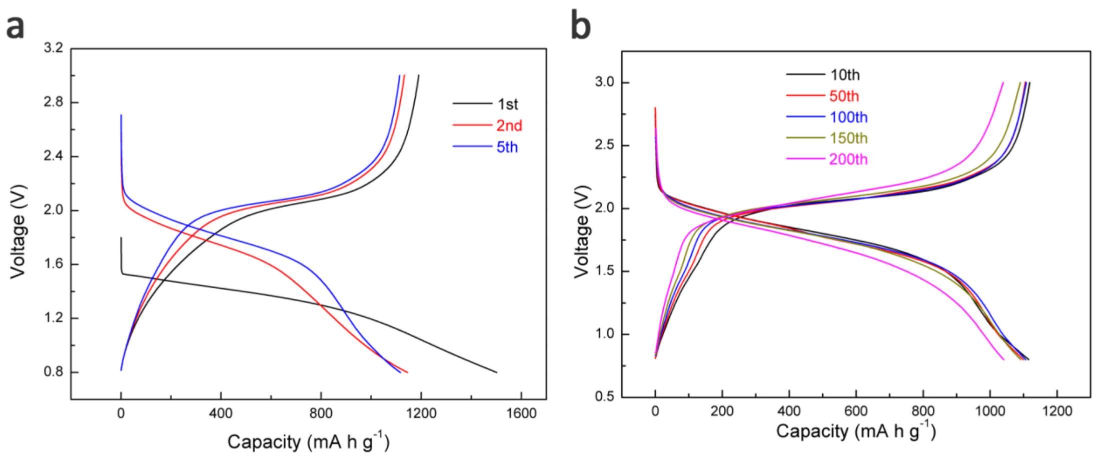

Figure S13. Typical discharge/charge profiles of sulfur-graphite full cells at different cycles with the NE-I electrolyte. a, The initial five cycles. **b**, The battery profiles at different cycles ranging from 10 to 200.

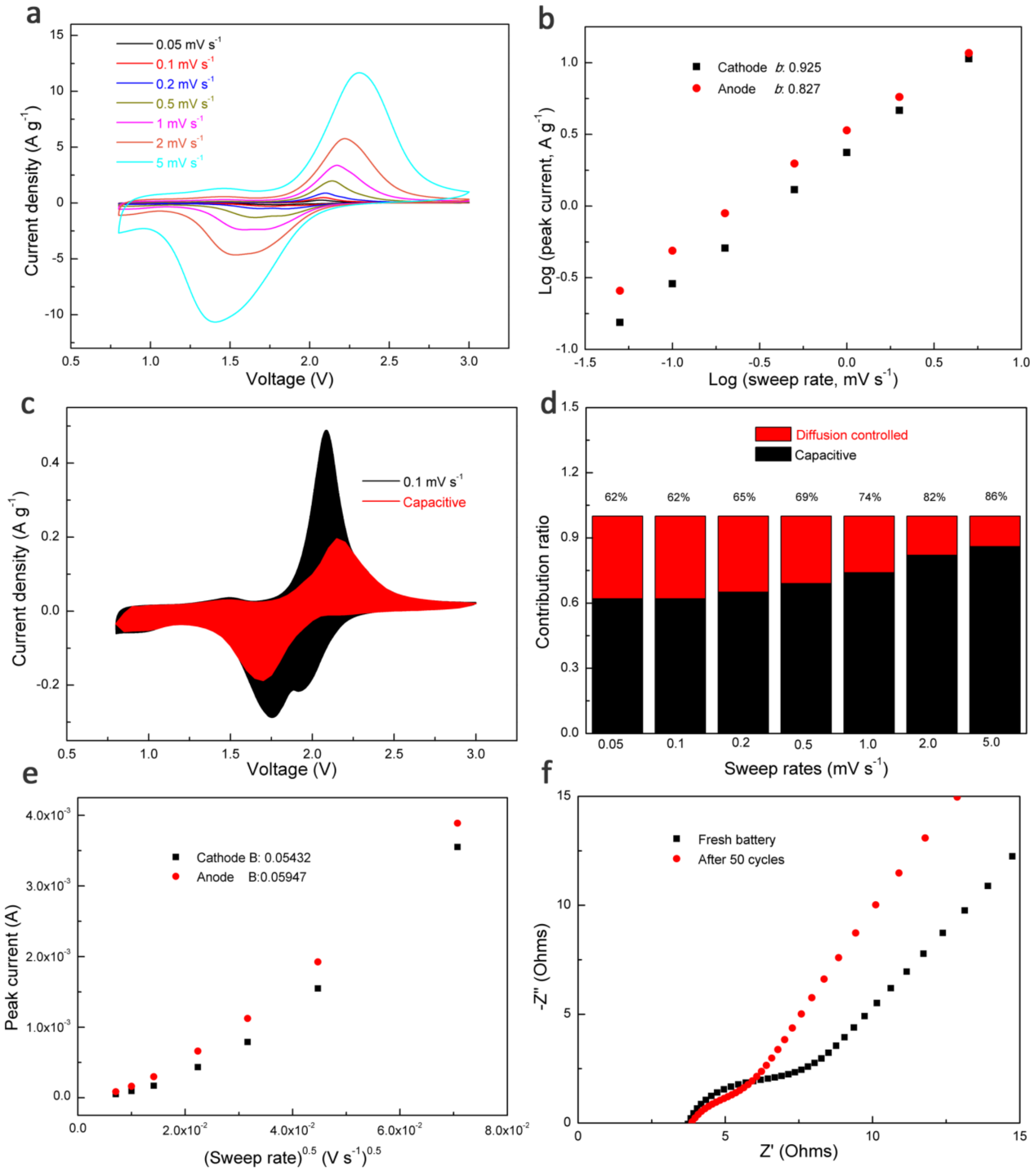

Figure S14. The kinetics analysis of a sulfur-graphite full cell with NE-I electrolytes. a, CV curves with various sweep rates. **b,** The *b* value of the full cell. **c,** The capacitive response at 0.1mV s^{-1}. **d,** The ratios of capacitive and diffusion-controlled responses at various sweep rates. **e,** The Li^+ diffusion coefficient kinetics analysis of the full cell. **f,** the EIS of the full cell before and after 50 cycles.

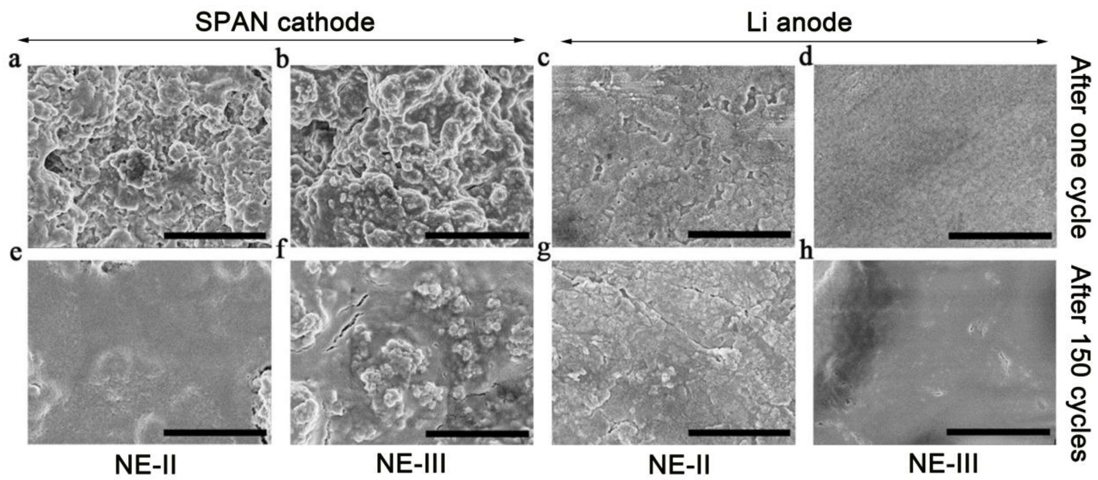

Figure S15. The SEM images of SPAN and lithium electrodes with NE-II and NE-III. a-d, After one cycle. **e-h,** After 150 cycles. **a, b, e, f,** SPAN cathode. **c, d, g, h,** Li anode. **a, e, c, g,** NE-II. **b, f, d, h,** NE-III. Scale bars: **a-h**, 10 μm.

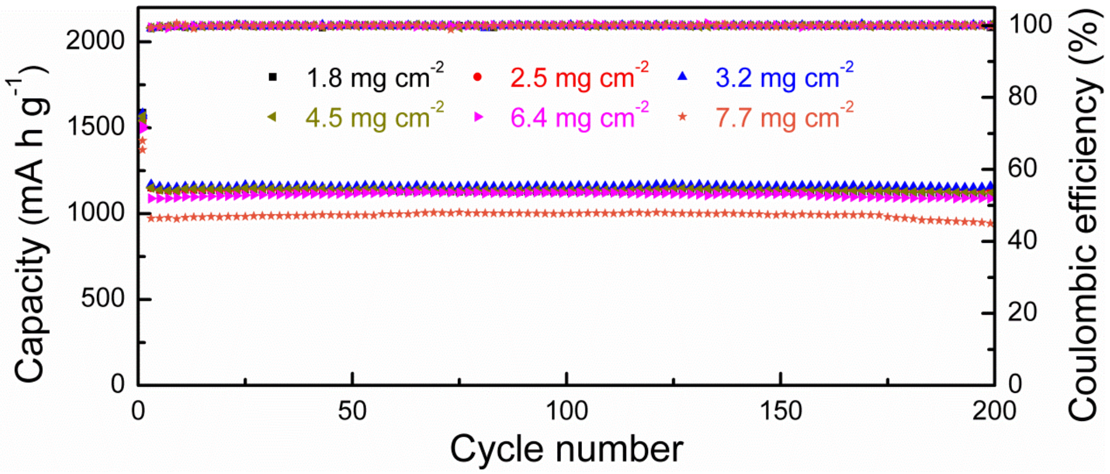

Figure S16. Cycling performance of batteries at a current density of 0.75 C with mass loading from 1.8 to 7.7 mg cm^{-2} with NE-II.

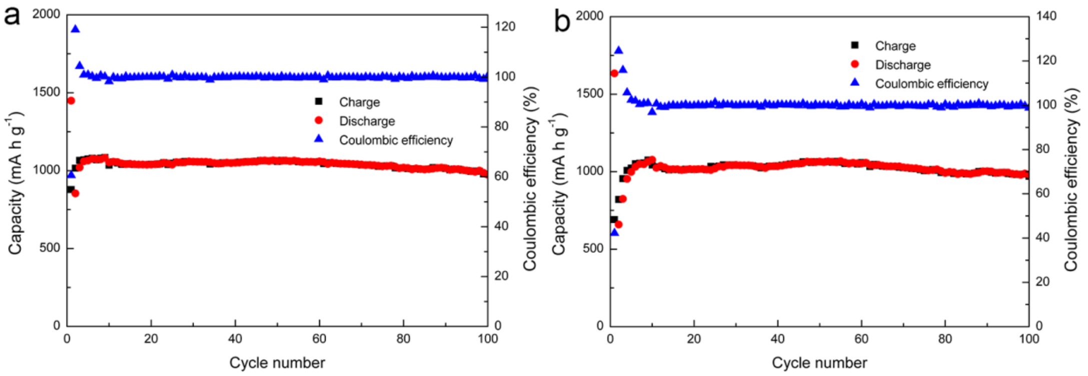

Figure S17. Cycling performance of batteries with an active materials loading range of 10-15 mg cm^{-2}. Pressure exerted on the electrodes at **a,** 0 MPa. **b,** 9 MPa. The electrodes were made of 90% active materials and 10% CMC.

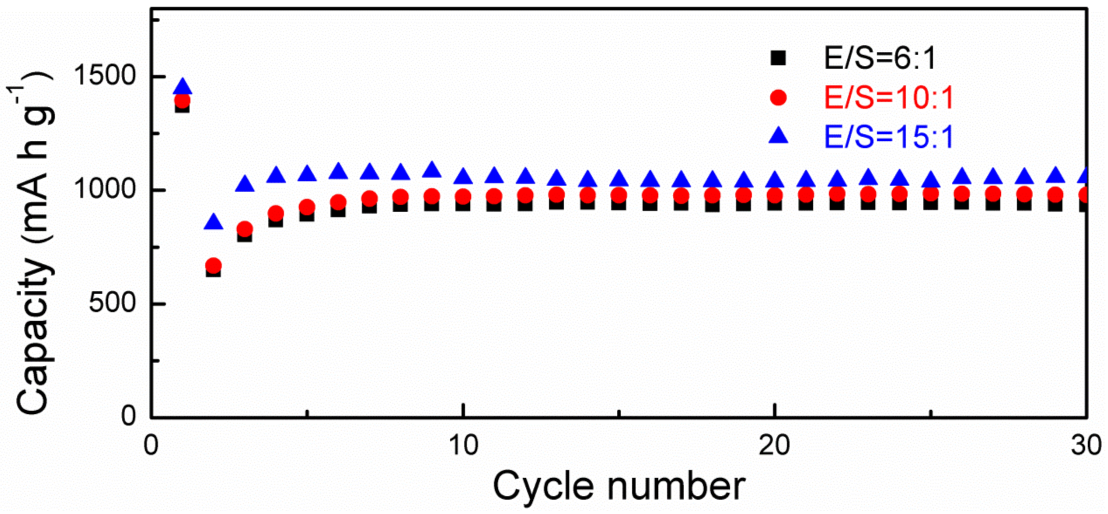

Figure S18. Cycling stability of SPAN cathode with different NE-II electrolytes: sulfur ratio (μL mg^{-1}) at 0.15C. The active materials loading range is 10-15 mg cm^{-2}.

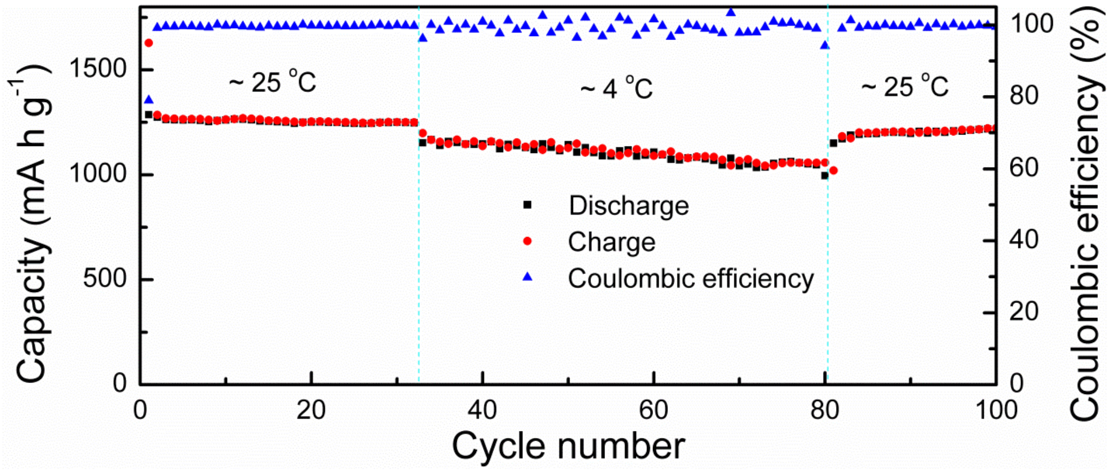

Figure S19. Cycling stability of batteries at a current density of 0.15 C at two temperatures.

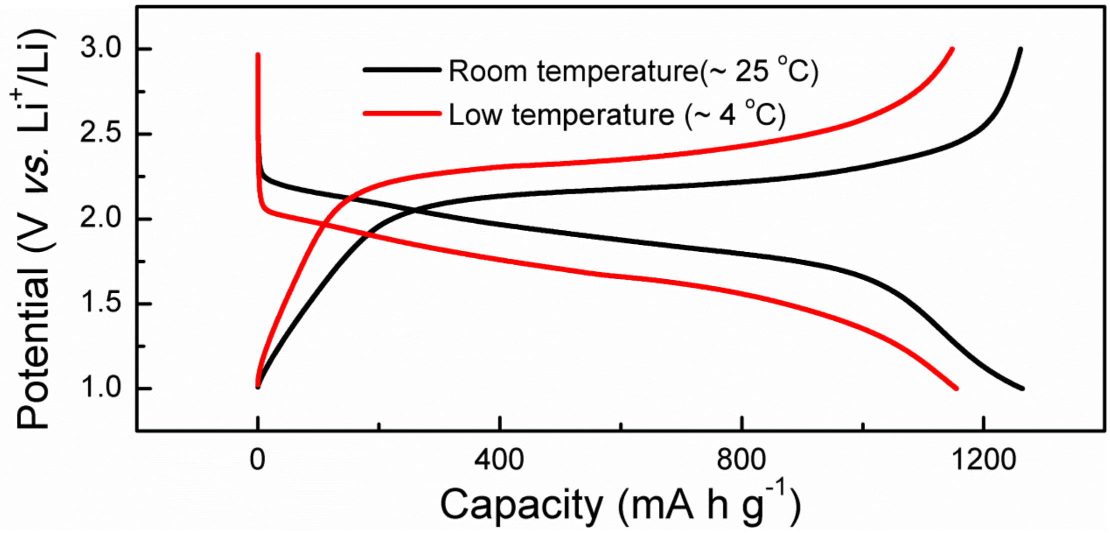

Figure S20. The typical charge/discharge profiles of the Li-S batteries at different temperatures.

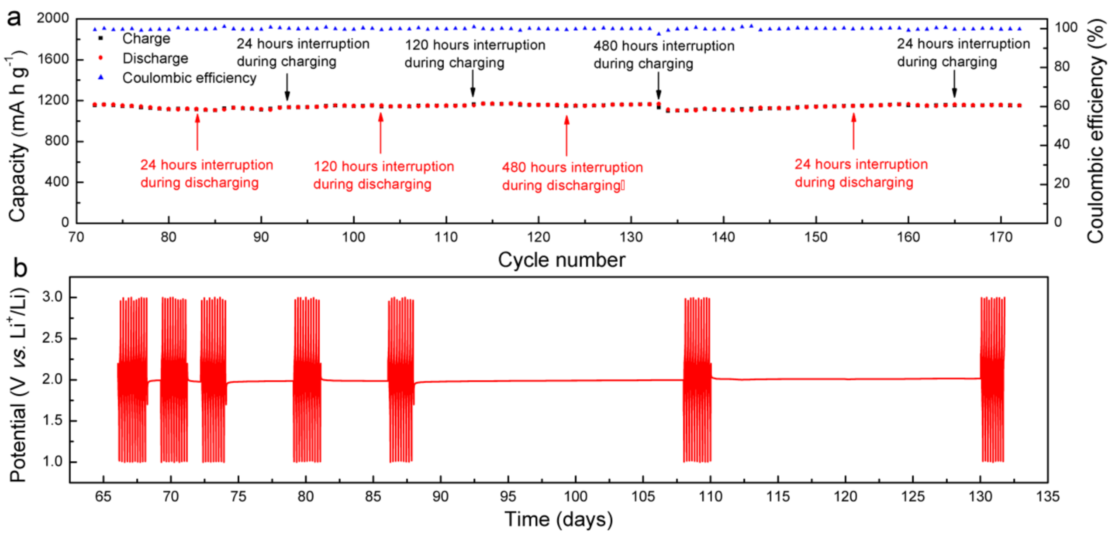

Figure S21. The storage tests of batteries. a, Storage for different hours during the charge/discharge process. **b**, Voltage-Time curve during the storage and cycling.

Table S1. Comparison of the rate capacity of the five electrolytes used in this study (unit: mA h g^{-1}).

	0.15C	0.3C	0.75C	1.5C	3C	7.5C
TE-I	1362	1120	904	753	524	198
TE-II	1287	1168	1092	1013	818	472
NE-I	1287	1192	1121	1094	885	655
NE-II	1393	1292	1238	1194	1038	750
NE-III	1430	1296	1232	1183	1015	703

Notes:

Note S1. Characterization of SPAN.

The SEM and TEM images indicated that SPAN is comprised of spherical particles with diameters range from 100 nm to 300 nm. The EDS mapping analysis of SPAN indicated a homogenous distribution of sulfur in the SPAN. The HRTEM image indicated the SPAN as polycrystalline, which was confirmed by the observation of rings in the SAED pattern. The XRD pattern of SPAN exhibits two typical peaks at approximately 25° and 39.3^{o} which corresponds respectively to the (002) and (100) planes of disordered carbon. The FTIR transmission spectrum of SPAN (obtained by embedding SPAN in a KBr pellet) indicated the presence of the following peaks at approximately 1492 cm^{-1} (C=C symmetric stretch), 1421 cm^{-1} (C=N asymmetric stretch), 1355 cm^{-1} (C-C deformation), 1227 cm^{-1} (C=N symmetric stretch), 933 cm^{-1} (ring breathing mode, side chain containing S-S), 795 cm^{-1} (ring breathing mode, main chain hexahydric ring), 660 cm^{-1} (C-S stretch), and 506 cm^{-1} (S-S stretch).[1] The C-S bond and the ring breathing mode with S-S side chains clearly substantiate the incorporation of sulfur into SPAN via covalent bonding. Furthermore, the Raman spectrum verified the existence of S-S (460 cm^{-1}) and ring stretch of S-S (951, 1102 cm^{-1}). The SPAN was also highly disordered, as indicated by the D (1320 cm^{-1}) and G bands (1511 cm^{-1}) with an I_D/I_G ratio of 1.05. Of the full survey XPS measurements performed to further elucidate the chemical state of SPAN, four distinct peaks located at 530 eV (O1s), 398 eV (N1s), 285 eV (C1s), 228 eV (S2s), and 163 eV (S2p) were observed. In the high-resolution C1s XPS spectrum, the three sub-peaks were assigned to sp^2 C-C bonds (284.5 eV), C-S bonds (285.4 eV), and C=N bonds (286.6 eV).[2] The high-resolution N1s XPS spectrum exhibits two typical peaks located at 398.0 eV and 399.9 eV, corresponding to the pyridinic N and pyrrolic N,

respectively. As for the high-resolution S2p XPS spectrum the main S2p 3/2 peak located at 163.2 eV was lower than that of elemental sulfur (164.0 eV), confirming the presence of C-S bonds. Another peak at 161.5 eV accounts for the adsorbed HS_xC, which is a byproduct formed during the sulfurization reaction. The peak at 167.8 eV, which corresponds to sulfate, is attributed to the oxidation of sulfur during the reaction. The existence of O1s peak is a clear indication for the oxidation of sulfur during the polymerization reaction, and the two sub-peaks at 530.46 eV and 531.88 eV of high-resolution O1s XPS correspond to the oxide and S-O, or S=O. This detailed characterization clearly indicated the incorporation of sulfur into SPAN through the formation of C-S or S-S covalent bonds.[3] The sulfur content is 41.43% as deduced from XPS analysis, which is consistent with the element analysis (41.15%), further indicating that the elemental sulfur is homogenously present in SPAN.

Note S2. The kinetics analysis of the sulfur-graphite full cell.

For convenience, kinetics analysis was used to elucidate the electrochemical properties of the full cell with electrolyte NE-I. By plotting the relationship between the log(peak current) and log(scan rate) of the CV curves of the full cell at various scan rates from 0.05-5 mV s^{-1}, the *b*-value of the cathode and anode of the full cell was calculated as 0.925 and 0.827, respectively, indicating that the main current in the peak is capacitive.[4] It is also possible to separate this current from the capacitive and diffusion-controlled processes. The total current and the capacitive current at the scan rate of 0.1 mV s^{-1} reveals the occurrence of diffusion-controlled process mainly at the peak, which is due to the reaction of S with Li^+. The results of the contribution ratios between the capacitive and the diffusion-controlled processes at various scan rates recorded an increase in the capacitive contributions with an increase in the scan rates.[4] The pseudocapacitance of the full cell is possibly from the Li^+ insertion into the graphite. Furthermore, the calculated average of this Li^+ diffusion coefficient of the full cell is 4.4×10^{-9} cm^2 s^{-1}, and the high Li^+ diffusion coefficient is beneficial for the electrochemical performance. The Nyquist plots of the full cell before and after the completion of 50 cycles yielded a very small diameter of the semicircle with the intercept indicative of the diminutive resistance of the charge transfer and the internal resistance, which accounts for the excellent electrochemical performance.

Note S3. The kinetics analysis.

Generally, an analysis of CV curves, via the equation $i = av^b$, is used to identify the contribution of these capacitive or diffusion-controlled processes.[4] The current i obeys a power law relationship with the sweep rate v, with a and b serving as the adjustable

parameters. The *b*-values are calculated from the slope by plotting log *i* *vs*. log *v*. In particular, the *b*-value of 0.5 represents a fully diffusion controlled process, while a *b*-value of 1.0 indicates a fully capacitive process. The ratio these contributions can be estimated accordingly from $i(V) = k_1 v + k_2 v^{0.5}$, where *i*(V) represents the current density at certain sweep rates, and k_1, k_2 are constants for a given potential. The k_1 is determined as the slope by plotting $i(V)/v^{0.5}$ versus $v^{0.5}$, so the ratio of the current due to the capacitive and the diffusion-controlled process can further be obtained by varying the sweep rates.

The Randles–Sevick equation $i_p = 0.4463nFAC(nFvD/RT)^{0.5}$ is used to estimate the Li^+ diffusion coefficient for evaluating the kinetics of the electrodes.[5, 6] By plotting i_p *vs.* $v^{0.5}$, which has a Y=BX form where $B = (269{,}000)n^{1.5}AD^{0.5}C$. Here, *n* represents the number of electrons transferred in the redox process and is equal to 2 in this case, *A* represents the electrode area (here A=1.13 cm^2), *F* is the Faraday constant (in C mol^{-1}), *D* represents the diffusion coefficient (in cm^2 s^{-1}) and *C* is the concentration of Li ions (here C=0.001 mol cm^{-3}).

References

S1. Wei S, Ma L, Hendrickson KE, Tu Z, Archer LA. *J. Am. Chem. Soc.* **2015,** *137*, 12143.

S2. Yan J, Li B, Liu X. *Nano Energy* **2015**, *18*, 245.

S3. Fan L, Ma RF, Yang YH, Chen SH, Lu BA. *Nano Energy* **2016**, *28*, 304.

S4. Zhu Y, Peng L, Chen D, Yu G. *Nano Lett.* **2016**, *16*, 742.

S5. Jung HG, Hassoun J, Park JB, Sun YK, Scrosati B. *Nat. Chem.* **2012**, *4,* 579.

S6. Wang J, Lin F, Jia H, Yang J, Monroe CW, NuLi Y. *Angew. Chem. Int. Ed.* **2014**, *53,* 10099.
